# Elucidating activation and deactivation dynamics of VEGFR-2 transmembrane domain with coarse-grained molecular dynamics simulations

**DOI:** 10.1371/journal.pone.0281781

**Published:** 2023-02-16

**Authors:** Yeon Ju Go, Mahroof Kalathingal, Young Min Rhee

**Affiliations:** 1 Department of Chemistry, Korea Advanced Institute of Science and Technology (KAIST), Daejeon, Korea; 2 Department of Chemistry, Pohang University of Science and Technology (POSTECH), Pohang, Korea; University of Helsinki, FINLAND

## Abstract

The vascular endothelial growth factor receptor 2 (VEGFR-2) is a member of receptor tyrosine kinases (RTKs) and is a dimeric membrane protein that functions as a primary regulator of angiogenesis. As is usual with RTKs, spatial alignment of its transmembrane domain (TMD) is essential toward VEGFR-2 activation. Experimentally, the helix rotations within TMD around their own helical axes are known to participate importantly toward the activation process in VEGFR-2, but the detailed dynamics of the interconversion between the active and inactive TMD forms have not been clearly elucidated at the molecular level. Here, we attempt to elucidate the process by using coarse grained (CG) molecular dynamics (MD) simulations. We observe that inactive dimeric TMD in separation is structurally stable over tens of microseconds, suggesting that TMD itself is passive and does not allow spontaneous signaling of VEGFR-2. By starting from the active conformation, we reveal the mechanism of TMD inactivation through analyzing the CG MD trajectories. We observe that interconversions between a left-handed overlay and a right-handed one are essential for the process of going from an active TMD structure to the inactive form. In addition, our simulations find that the helices can rotate properly when the overlaying structure of the helices interconverts and when the crossing angle of the two helices changes by larger than ~40 degrees. As the activation right after the ligand attachment on VEGFR-2 will take place in the reverse manner of this inactivation process, these structural aspects will also appear importantly for the activation process. The rather large change in helix configuration for activation also explains why VEGFR-2 rarely self-activate and how the activating ligand structurally drive the whole VEGFR-2. This mechanism of TMD activation / inactivation within VEGFR-2 may help in further understanding the overall activation processes of other RTKs.

## Introduction

Receptor tyrosine kinases (RTKs) control many cell activities such as growth, migration, survival, proliferation and differentiation [1]. Among RTKs, vascular endothelial growth factor receptor (VEGFR) regulates angiogenesis that plays a crucial role in embryogenesis and organ development [2–4]. VEGFR is composed of an extracellular ligand-binding domain (ECD), a single transmembrane domain (TMD), and an intracellular kinase domain (ICD). It is known that receptor dimerization and receptor rearrangement are essential for signal transduction in VEGFR [3,5]. When a ligand is attached to a pre-dimeric form of VEGFR [6], the receptor rearrangement is promoted [5,7]. ECD rearrangement promotes TMD helix rearrangement and triggers ICD activation [5,8]. Therefore, the proper rearrangement of TMD plays an important role in the activation of VEGFR.

**Fig 1:**
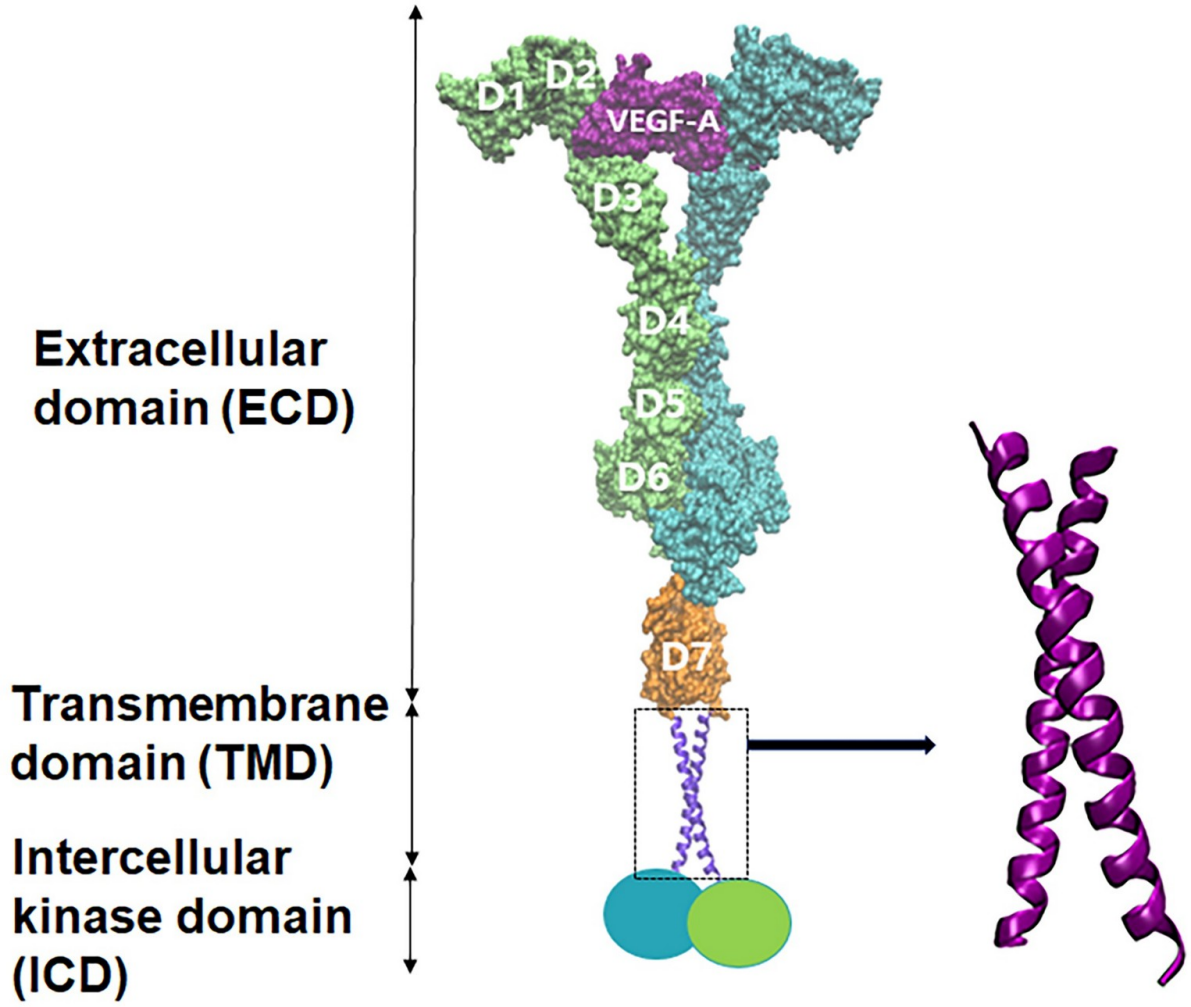
Schematic illustration of the ligand-bound VEGFR-2 dimer structure. The VEGFR-2 dimer is composed of ECD with seven subdomains, TMD through lipid bilayer, and ICD in cytoplasm. The magnified structure of TMD on the right is based on an experimental structure (PDB ID: 2M59).

There are a few different types of VEGFRs with varying functions. Among these, VEGFR-2 works as a primary regulator of endothelial migration and proliferation [9] and has been a focus of many continuing studies [10–12]. In VEGFR-2, the signal transduction of extracellular stimuli to the cytoplasm (Fig 1) is achieved by the allosteric and oligomeric conformational change of TMD [13]. Research over the past decades has proven that TMD needs to have dimerized helices for receptor activation, and that the rotations of the two helices around their own axes at specific angles are essential for the activation of the ICD part linked to TMD [5,8,14,15]. Indeed, studies on how rotational changes in TMD affect the receptor activation in various RTKs have demonstrated certain preferences of specific orientations of TMD toward the activation [8,13,16–20]. In the case of VEGFR-2, a mutation study elucidated that each of the dimeric helices of TMD rotates by 180 degrees when VEGFR-2 converts from its inactive state to the active state [7]. In this study, VEGFR-2 with the G770E/F777E double mutant TMD was always activated with or without the ligand. In addition, each helix was rotated by 180 degrees relative to the interface of the WT TMD structure that corresponds to the inactive state, suggesting that after ligand binding to the ECD part, the TMD helices will likely rotate 180 degrees relative to the inactive TMD conformation. However, because the structure was fixed by the mutation, the detailed process over which the TMD dimer will pass from the inactive state to the active one could not be elucidated. For revealing the process, adopting computational tactics will be helpful. Indeed, there have been tremendous recent advancements in various in-silico techniques and strategies to overcome the limitations of experiments [21–32]. Among the diverse approaches, for following how the TMD dimer changes in time, adopting molecular dynamics (MD) simulations will be a natural choice. Actually, all-atom (AA) MD simulations were also adopted and revealed that the inactive WT-TMD structure of VEGFR-2 is stable over several hundred nanoseconds [7]. Because VEGFR-2 is a rather large system with multiple domains, however, it will be desirable to reach much longer simulation time scales.

Here, we have performed such long-time simulations of WT-TMD with the help of coarse grained (CG) MD simulations [33–40] for uncovering activation / deactivation mechanism of TMD. In fact, CG MD simulations have been used to investigate the dynamics of RTKs or their TMD parts [41–45]. We have employed milliseconds of CG MD simulations using the MARTINI force field as it is hundreds of times less burdensome than AA MD and can still display acceptable reliability [46,47]. In particular, the MARTINI force field model was already used toward observing some events such as helix rotations and crossing angle changes [42], similarly to what we are interested in. We also produced the free energy surface (FES) to identify the meta-stable structures of the TMD part of VEGFR-2 from our CG MD simulations. Through analyzing this FES, several inactive structures of TMD are located. In addition, we observed that the simulations that started from the inactive TMD structures stayed in the same region stably over a relatively long time. This suggests that TMD may not independently and spontaneously change its shape from an inactive form to an active one without the help of an external force induced by ECD with its ligand. Namely, the stability of the inactive TMD structure may be the reason why VEGFR-2 requires a ligand for activation [5–8]. Thus, to access the TMD activation mechanism, we have instead attempted inactivation simulations by starting from an experimentally resolved activated conformation. This reversed tactic is philosophically based on the principle of microscopic reversibility, and has often been applied to studying protein folding [7,48,49]. Through our simulation results, we reveal that the inter-helix conformational changes are strongly correlated with the helix rotations around the helical axes. More specifically, the two helices of TMD need to take a pivoting motion involving a significant change in the crossing angle, and interconversions between a left-handed structure and a right-handed one were often detected. These changes are essential toward initiating helical rotations that are directly related to the VEGFR-2 activation. Our computational strategy may also be applicable for studying other RTKs.

## Materials and methods

### Coarse grained simulation protocol

**Fig 2:**
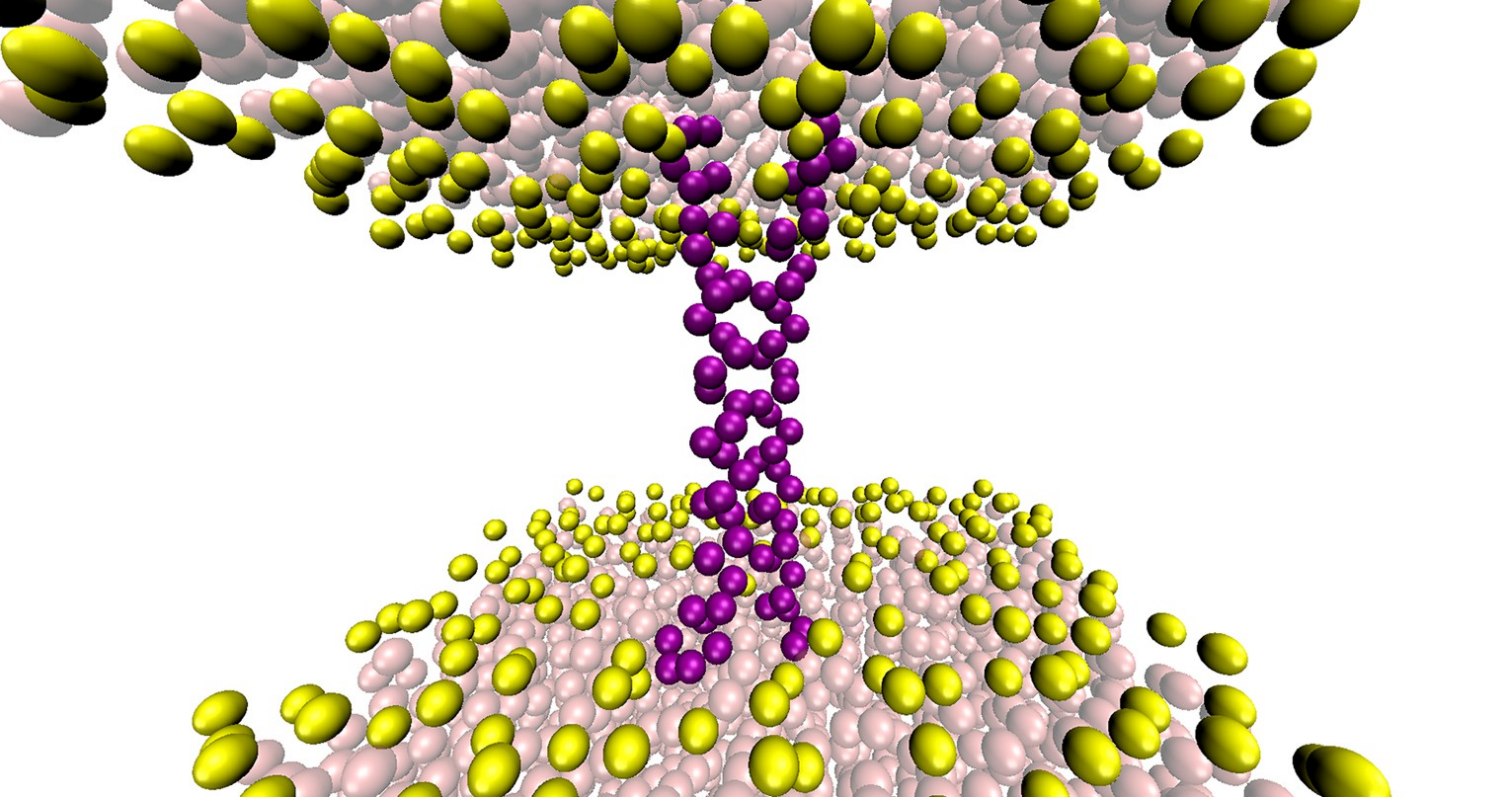
The structure of the initial system used in CG MD simulations. TM helix dimer buried in lipid bilayer is shown in purple. The CG beads of phosphate and choline groups of POPC lipid bilayer are shown in yellow. Standard MARTINI water particles are shown as transparent beads.

All CG MD simulations were performed using GROMACS [50]. MARTINI 2.2 force field [46] was used to convert the atomistic TMD structure known as active conformation to coarse grained representation. This initial TMD structure was made using the WT sequence of VEGFR-2 TMD and using the one known as the TMD active structure (PDB ID: 2MEU) [7] as a template with SWISS-MODEL [51]. The adopted parameters for the model evaluation were: Global Model Quality Estimate [51], 0.63; QMEANDisCo global score [52], 0.54 ± 0.11; QMEAN Z-score [53], −2.15; QSQE [54], 0.36; and sequence similarity, 0.56. The xssp 3.0.8 version [55,56] was used for dssp program to determine the secondary structure of the protein backbone. TM helix dimer was inserted within a single lipid bilayer consisting of 1-palmitoyl-2-oleoyl-*sn*-glycero-3-phosphocholine (POPC) containing 187 lipid molecules using INSert membrane tool [57]. TMD and lipids were solvated with 2887 standard MARTINI water particles, and 0.15 M of NaCl was added while neutralizing the system (Fig 2). The initial system was energy minimized using the steepest descent method. The minimization convergence was declared when the maximum force was smaller than 100 kJ mol^−1^ nm^−1^ (in 704 steps). After this, 25 systems with different initial velocities were equilibrated for 20 ns to relax the solvent and complex lipid bilayer around TMD, producing 25 different TMD systems for production MD. At this step, position restraints were applied to all residues of TM helix dimer with a force constant of 1000 kJ mol^−1^ nm^−2^. Unrestrained production simulations were then performed in NPT ensembles such that 25 production CG MD simulations were run for 100 μs each using 40 fs integration time steps. The temperatures of the protein, lipid bilayer, and solvent were maintained at 323 K using the velocity-rescale thermostat [58] with the relaxation time of 1 ps. The pressure was semi-isotropically coupled at 1 bar employing the Parrinello-Rhaman barostat [59] with the coupling constant of 10 ps and compressibility of 3×10^−5^ bar^−1^. Lennard-Jones interactions were shifted to zero between 0.9 to 1.2 nm and electrostatic interactions were shifted to zero between 0 and 1.2 nm with a relative dielectric constant of 15. Neighbor lists were updated every 20 steps using 1.2 nm cutoff.

### Analysis

The simulation results were analyzed using GROMACS [50], VMD [60], residue-residue contact score (RRCS) [61], and home-built scripts. In order to calculate RRCS of TMD, AA TMD structures were first attained by back mapping [62] the CG TMD structures obtained every 4 ns in the CG MD simulations. After this, the RRCS was calculated for each AA TMD structure to find interhelical residue pairs between the two TMD helices. In the case of calculating free energies and analyzing TMD structures, the adopted variables were lateral helix separation (*L*) between the TM helices, crossing angle (Ω) between TM helices, the difference in interhelix distance at the N-termini and the C-termini (Δ*d*) and the rotation of the helices along their long axis relative to each other. The lateral distance between TM helix monomers is calculated using GROMACS analysis command, gmx pairdist. The crossing angle (Ω) and the rotational angle of TM helix monomers were calculated by homemade scripts. The absolute value of the crossing angle was decided using the definition |Ω|=acos(h1⋅h2/|h1||h2|). Here, Ω is less than 90 degrees, and *h*1 and *h*2 are eigenvectors of helix A and helix B. The negative sign of Ω means a left-handed helix while the positive sign means a right-handed one. The rotational angle of each helix is obtained by averaging the rotational angles of residues about each helix axis for all residues. Detailed calculations are described in the next part of the method. The interhelical contact residue pairs used to analyze the concordance rates of the two TMD structures were obtained using RRCS. All figures with molecular structures were rendered using VMD.

### Analyzing rotational angle of the helix

**Fig 3:**
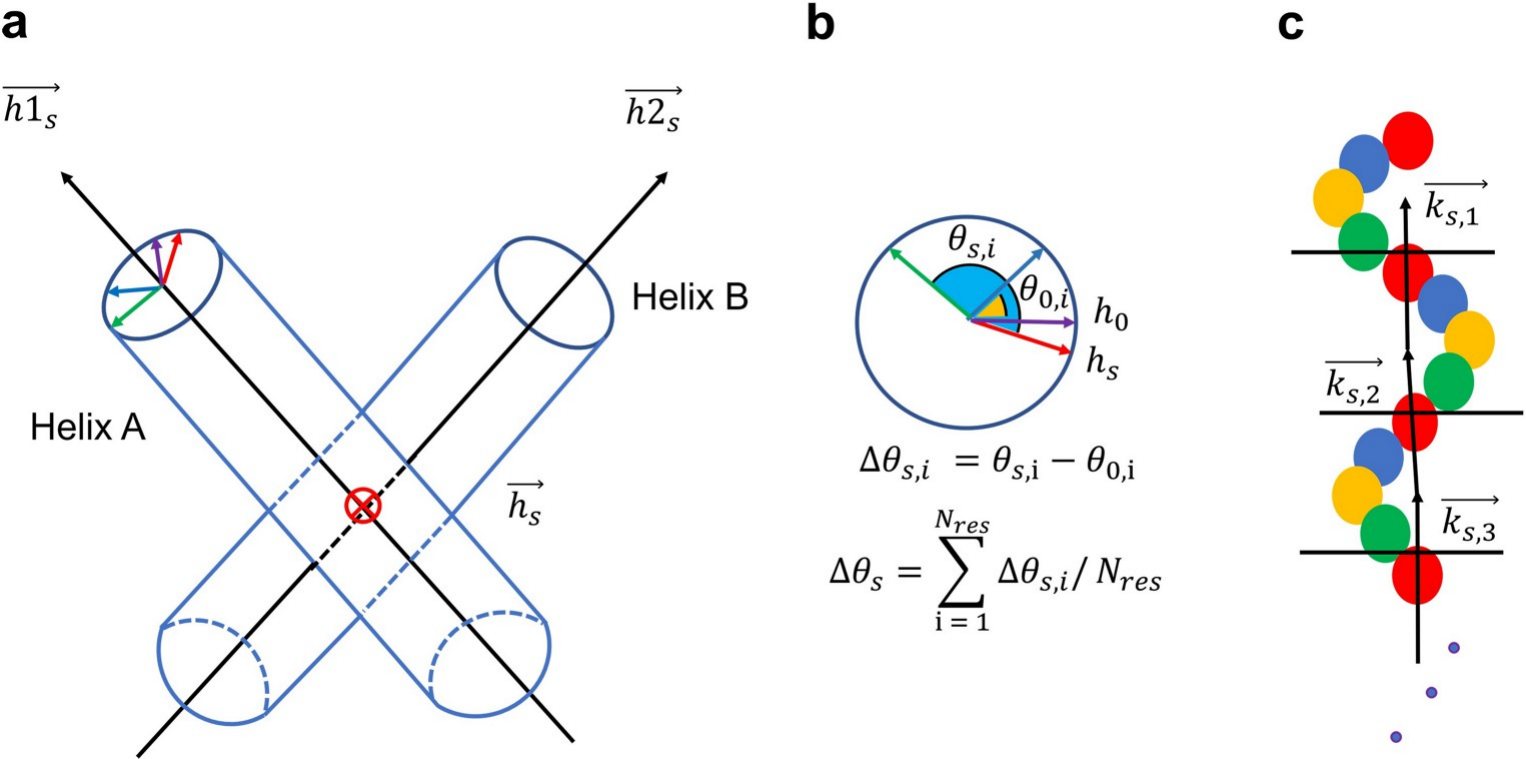
Schematic illustrations of the strategies for defining helix rotational angles. (a) Axes of helix A and helix B, and the cross product of the two axes as the reference for rotation. (b) Definitions of residue-specific rotations and the final helix rotation. (c) Definitions of {*k*_*s*,*j*_} for handling helix bending and wiggling.

A helix rotational angle was defined as the average of the rotational angles of all backbone of residues. All residues corresponding to the backbone of VEGFR-2 TMD were counted by how many degrees they were rotated, with respect to the axis of the helix they belong to. For this, we took a convention of taking the rotation angle of the TMD helix as positive when it rotated in a counterclockwise manner. One TMD structure is composed of helix A and helix B, and the axis of each helix is made of a vector that passes the center point of the four backbone residues at the leading part of the TMD sequence and the center point of the backbone residues at the trailing part of the TMD sequence. The vector *h*_*s*_ (*s* = 0,…,*N*_frame_) that is parallel to the cross product of the direction vectors of helix A (*h*1_*s*_) and helix B (*h*2_*s*_) was used as a reference line for calculating the rotation angle of any residue (Fig 3A). Here, *N*_frame_ designates the number of trajectory snapshots employed for the analysis. The direction of *h*_*s*_ was determined so that it always pointed from the helix we wished to calculate the rotation angle to the other helix. However, due to the bending of the helices, when the crossing angle of helix A and B was too small, the helix rotation angle could not be reliably measured with the reference line defined in the above. To circumvent this issue, when the crossing angle was between −14.4 and 14.4 degrees, a midpoint of a helix was first defined as the center of mass of the residues 777–780 that are located in the middle of the helix, and a vector was projected from this midpoint to the other helix such that this vector becomes perpendicular to the axis of the partnering helix. The amount of change in the rotation angle of the *i*-th residue (*i* = 1,…,*N*_res_) at the frame index *s* was obtained by subtracting the initial rotation angle, namely Δ*θ*_*s*,*i*_ = *θ*_*s*,*i*_−*θ*_0,*i*_. The helix rotation of TMD at the *s*-th frame (Δ*θ*_*s*_) can be obtained by averaging these changes over all residues (Fig 3B). When obtaining the rotational angle, it was necessary to also consider the fact that the TMD helices tended to bend and wiggle, and we adopted the following strategy as a remedy. First, the TMD sequence was divided into groups of four residues (Fig 3C), and the group-specific center points were calculated. Then, the direction vectors that pass through two neighboring center points were obtained as *k*_*s*,*j*_ ($j=1,\ldots ,\frac{1}{4}N_{\mathrm{res}}^{\prime }-1$). In TMD, there are *N*_res_ = 25 residues, and we included three additional residues from the linker region trailing to ICD to set as $N_{\mathrm{res}}^{\prime }=28$. Note that there are 7 center points and 6 direction vectors. To find the rotation angles of the four residues forming the first group at the N-terminus, *k*_*s*,1_ was used as the axis, and the projecting line from one residue to the axis was employed toward calculating the residue-specific rotational angle. In the same manner, the rotation angle of the residues in the *j*-th group was found by referring to projections on to *k*_*s*,*j*_ up to *j* = 6. Finally, the rotation angle of the final 25th residue was obtained by adopting *k*_*s*,6_, as there is no direction vector *k*_*s*,7_ defined. Of course, the reference vector *h*_*s*_ was purified by projecting out its component along *k*_*s*,*j*_ in this case.

### Free energy landscape

Free energy landscapes were calculated based on TMD structures obtained from CG MD simulations, mainly as functions of TM helix rotational angles. The free energy was obtained by *U*(*x*) = −*k*_*B*_*T* ln *p*(*x*), where *p*(*x*) is the histogram estimate of the probability density of *x* in the simulated trajectories with *k*_*B*_ and *T* denoting the Boltzmann constant and temperature [63].

## Results and discussion

As explained in an earlier part, to elucidate the mechanism of rather slow and rarely occurring activation of TMD in VEGFR-2, we explored its inactivation dynamics. Experimentally, it was found that when ECD of VEGFR-2 was removed, the VEGFR-2 activity increased [7]. This was because ECD hinders the receptor activation in the absence of activating ligand, which has been known earlier for VEGFR-2 [7], EGFR [64], and FGFR [65]. It was also shown that ICD limited receptor activation [1]. With these, one might expect that the active TMD conformation should be observed when simulations are performed with only TMD after truncating ECD and ICD. However, higher activity does not necessarily mean that the activated form of TMD is more stable than the inactive form. Indeed, when we initiated simulations from the inactive TMD structure, conversion into any stable and long-lasting active conformation was not observed (S1 Fig in S1 File). From this result, it can be inferred that the active TMD with truncated ECD and ICD is structurally unstable. At the same time, the ligand is essential for TMD to become active. This was the reason we decided to observe the inactivation mechanism first by adopting the active TMD conformation as the initial structure of simulations with CG MD. With the principle of microscopic reversibility [48,49], the reverse of the inactivation process should well represent the activation mechanism. In fact, the principle of reversibility has often been applied to studying relatively slowly folding proteins, where the reverse of relatively fast unfolding was adopted for elucidating the folding process [66,67]. Based on the fact that backbone motions such as bending, twisting, and stretching of TMD helices can occur from microsecond to millisecond time scales [7,8,68], an aggregate simulation length of 2.5 ms was applied. Considering that motions in CG MD tend to be faster than the equivalent motions in all-atomistic MD [47], this should be long enough for the given purpose.

In addition, to verify that our CG MD scheme works well, we tested whether a mutant TMD (PDB ID:2MEU) experimentally known to be active by itself [7] correctly displayed the structural characteristics found in experiment. For this, we generated an inactive form of this mutant (S2 Fig in S1 File) by enforcing it to follow the WT-TMD structure and performed the same CG MD simulation. We found that it changed well into the active structure (S3 Fig in S1 File), consistently with the experimentally found one [7].

### Free energy surface

A total of 25 production simulations were performed by varying the initial velocity with a common starting structure representing the TMD active state. Trajectories of 100 μs duration were generated using the simulation protocol described in Methods using 40 fs integration time steps. Coordinates were saved at every 400 ps. For the activation of the receptor, two TM helix monomers should dimerize [69–72] with each helix monomer rotating at a specific angle in the same direction [5,73]. Because we are only interested in the dimerized activated TMD, only the TMD structures with lateral helix separation of *L* = 3 nm or less were taken for constructing the free energy profiles. TMD can exist in three states: monomeric (*L* > 3nm), pre-dimeric (1.5 nm < *L* < 3 nm), and dimeric (*L* < 1.5 nm). We constructed the free energy profiles as a function of rotational angles of the two TM helix monomers as defined in Fig 3. As a caution, we stress that the figure does not represent a statistically true free energy surface because our simulations only covered non-equilibrium behaviors, with the lacking inactive to active transformations. However, the clustering behaviors by the TMD conformations based on the helix rotation angles can still present meaningful information at least in the qualitative sense. While one may consider adopting a more rigorous approach such as the Markov state model [74–76] for generating a true free energy information, the relative crudeness of the CG model would not warrant the complications on the added simulations. In any case, the free energy profile of each CG MD trajectory was constructed separately. Indeed, the resulting 25 profiles revealed some common aspects.

**Fig 4:**
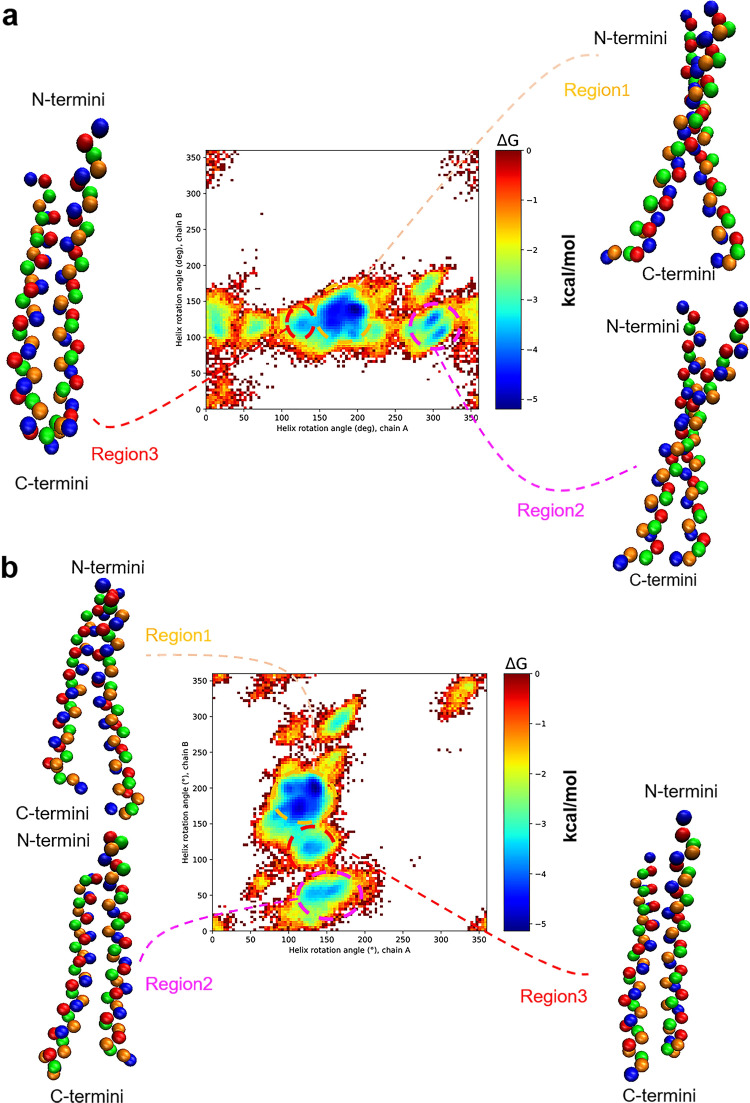
Free energy profiles and the representative simulated structures of the TM helix dimer. (a) The free energy profile and the simulated structures of the TM helix dimer from an arbitrarily chosen trajectory. Three basins appearing as free energy minima corresponding to inactive TMD structures are shown with dashed lines, together with their representative structures. TMD backbones are displayed with colored beads. (b) The same as in (a) but from another arbitrarily chosen trajectory.

Some representative TM helix dimer structures corresponding to the free energy minima are shown in Fig 4. FES from one trajectory, shown in Fig 4A, displays roughly three regions of frequently visited conformations. The first region shows the lowest free energy of −5.19 kcal/mol with the helix A rotational angle of about 160–200 degrees and the helix B rotational angle of about 100–150 degrees (denoted with the orange dashed line in Fig 4A). The average values of the crossing angle, *L*, and Δ*d* values of the TMD structures in this region are −27.1 degrees, 1.03 nm, and −0.3 nm, respectively. To find out the interhelical residues toward forming the structures in this region, we calculated RRCSs, which showed that I766, I767, T771, I774, A775, F778, W779, and V783 were mainly observed in the interhelical contacts. In addition, TMD inactivation often involved helix sliding. For example, in many structures, I766 of helix A and T771 of helix B strongly formed an interhelical contact, and on average there was ~4.7 sequence difference between the contacting residues from the two chains. Because the starting active form was symmetric, this suggests that sliding motion also accompanies the inactivation. In the second region, helix A has rotated about 280–320 degrees and helix B has rotated about 100–140 degrees with −3.97 kcal/mol of a free energy change (denoted with the magenta dashed line in Fig 4A). This is equivalent to rotating helix A by 40–80 degrees clockwise, meaning that helix A here rotates less than in the first region. By analyzing RRCSs, we found that I766, L768, A775, W779, L781, L782, and I784 were mainly involved for the contacts in this case. In the third region, both helix monomers have rotated about 110–150 degrees with the free energy change of −3.68 kcal/mol (the red dashed line in Fig 4A). The averages of the crossing angle, *L*, and Δ*d* for the conformations in this region were −13.5 degrees, 1.00 nm, and 0.004 nm, respectively. Also, we found that these structures were similar to the NMR structure of inactive TMD [7]. In addition, RRCS analysis revealed I767, L768, T771, F778, W779, L781, L782, and I785 as the interhelical contact points.

Such frequently visited regions along inactivation actually change depending on the trajectory. For example, FES in Fig 4B drawn with another trajectory, still shows three representative free energy minima regions but at different angle values. Interestingly, the most frequently visited regions in Fig 4A and 4B are in a transposing relation with each other. Considering that the activated TMD structure is symmetrical, it can be explained that the inactivation breaks the symmetry and there are two ways of breaking it. Indeed, among the 25 FESs, the two ways were found to be almost equally probable. Moreover, when all the FESs are averaged into one final FES, the distribution appears quite symmetric (S4 Fig in S1 File). Thus, we can infer that inactivation leads to two dominant symmetry broken conformations together with additional local minimum states that are transiently visited. The transiently visited states are quite diverse and appear differently for different trajectories, and after averaging over multiple trajectories their free energy basins get washed out (S4 Fig in S1 File). Because the energy differences between inactive TMD regions in Fig 4A or in Fig 4B are only ~1 kcal/mol or even smaller, interconversions from one inactive form to another will be easy.

In Fig 4, a free energy basin is barely seen around the active TMD structure, and it can be inferred that the active TMD will be energetically quite unstable and thus maintaining the structure for a long enough time naturally without ligand binding will be difficult. Recall that the helix rotation angles are measured with respect to the active structure, and the point at (0, 0) in Fig 4 marks the active structure. Interestingly, the NMR structure of inactive TMD is close to the region 3 structure in Fig 4 (Table 1). In addition, the nearby regions exhibit rotational angles that are not too far from region 3 values, suggesting that our simulated structures in that region are close to the NMR structure. The most significant difference between the NMR data and our simulated structure is the sliding motion observed only in our simulations. This discrepancy may be related to the use of rather small micelles in the NMR experiment [7], which will hinder sliding motions. In any case, considering the stability of the inactive form of TMD, the probability of false signaling by VEGFR-2 in normal situations will be very low. This is also consistent with the commonly accepted fact [1–4] that the conformational change produced by ligand binding to ECD is a prerequisite to VEGFR-2 signaling.

**Table 1:** Comparison of the NMR and the simulated structures of the inactive TMD form^a^.

	from NMR	from simulation
Lateral helix separation (nm)	0.96	0.85 ± 0.03
Crossing angle (deg)	−25.15	−21.90 ± 1.61
Helix A rotation (deg)	108.75	151.49 ± 6.78
Helix B rotation (deg)	110.69	139.13 ± 6.27

^a^ The processes of obtaining the values in this table are described in a later part, titled “Structural diversity of the inactive form”.

### Inactivation and activation mechanism of TMD

**Fig 5:**
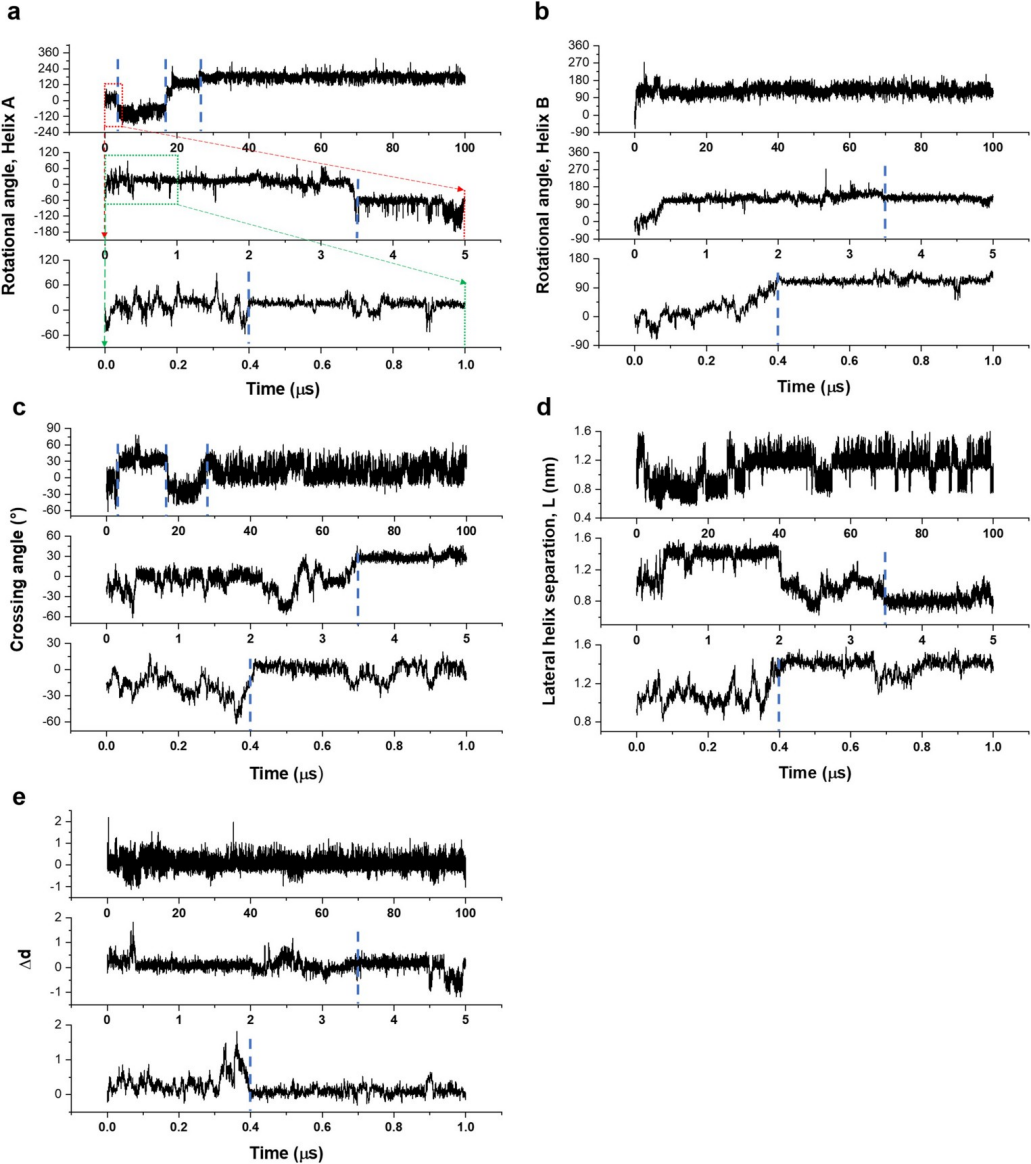
Analyses of collective variables of helix A and helix B in time. Time evolutions of (a) the helix A rotation angle, (b) the helix B rotation angle, (c) the crossing angle of TMD, (d) the lateral helix separation, and (e) Δ*d*. For visual clarity, three different time scales of 100 μs, 5 μs, and 1 μs have been employed for the horizontal axes in magnifying manners. Visual guides for the magnifications are schematically drawn in (a).

**Fig 6:**
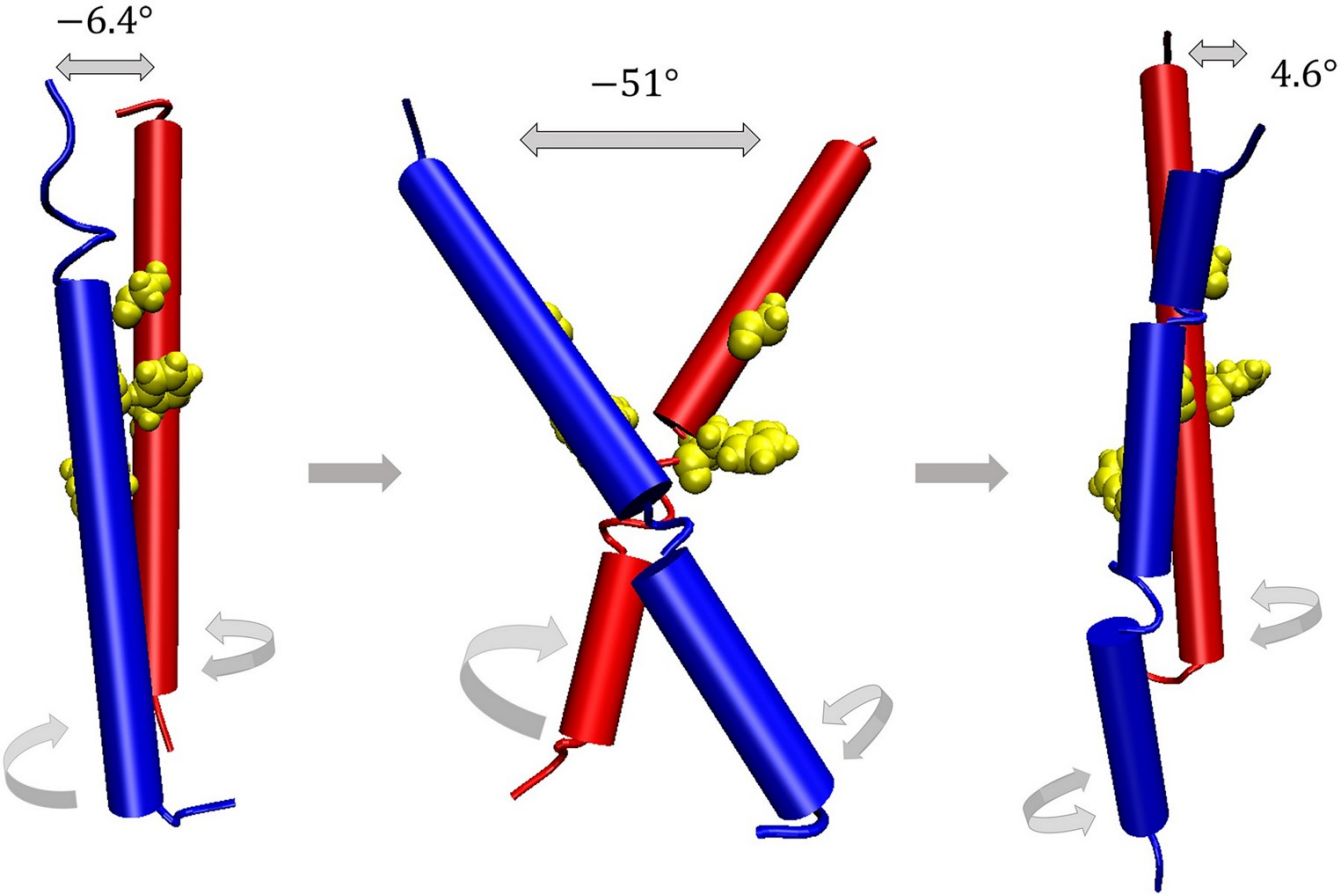
Schematic illustration of the inactivation mechanism. The blue / red cartoon representation designate helix A / B. TMD shows a pivoting motion during which helix B rotates. The sidechains of G770 and F777 are also shown in yellow.

When the 25 trajectories obtained from the CG MD simulations were analyzed using the crossing angle between helix A and helix B, the rotational angles of helix A and helix B, the lateral helix separation, and Δ*d* defined as the difference in interhelix distances at the N-termini and the C-termini, a common inactivation mechanism was observed from the majority of the trajectories. The data from one arbitrarily chosen trajectory are pictorially shown in Fig 5. Basically, because the active TMD conformation was very unstable, it readily changed to the inactive TMD structures. Analyses of the rotational angles of helix A and helix B in time (Fig 5A and 5B) found that a significant change in the helix B rotational angle occurred up to 0.4 μs, and this changed value remained fairly stable up to the end of the 100 μs simulation (Fig 5B). Meanwhile, around 3.5 μs, the helix A rotated by more than 50 degrees in the clockwise direction, and the TMD structure entered the FES local minimum for the first time (Fig 4A, region 2). First, focusing on the 0.26–0.4 μs section, where TMD satisfied the helix B condition for going to the FES minimum, the crossing angle changed from −6.4 degrees to −51 degrees and then to 4.6 degrees (Fig 5C), showing a clear pivoting motion (Fig 6). In addition, it was observed that the lateral helix separation increased over this time period from 1.0 nm to 1.4 nm (Fig 5D). Also, as the absolute value of the crossing angle increased, Δ*d* increased such that the distance between the two N-termini was larger than the distance between the two C-termini. Specifically, the difference between the two distances ranged from 0.0 to 1.5 nm (Fig 5E). Similarly, when the absolute value of the crossing angle decreased, Δ*d* also decreased for shorter N-termini distance. After 0.4 μs, namely, after TMD entered the FES minimum, Δ*d* returned back to its earlier value at 0.26 μs and before (Fig 5E). Second, analyzing the TMD dynamics around 3.5 μs where TMD satisfied the helix A condition for going to the FES minimum, the crossing angle changes from 4.6 degrees to −44 degrees and then to 27 degrees. Overall, we stress that the pivoting motion appears to be important when the active TMD structure changes into the inactive form, and the lateral helix separation and the N- and C-termini distances of the two helices change accordingly.

Because the free energy difference between different minima regions of the inactive TMD is rather small, TMD should be able to easily move between the minima. We indeed observed such structural changes over time. For example, during 16–26 μs, TMD moved from region 2 to region 3 and the helix A rotational angle changed roughly from −60 degrees to ~150 degrees. At this time, the distinct pivoting motion was again observed. During that time period,the crossing angle changed from 32 degrees to −28 degrees and then to 8.7 (Fig 5C). The lateral helix separation and the difference in interhelix terminal distances changed similarly to what was observed at the earlier time.

Similarly, after 26 μs, the rotational angle of helix A changes from 150 degrees to 200 degrees (Fig 5A), and the system moved from region 3 to region 1 in Fig 4A. This was accompanied by a significant change in the crossing angle, which even lasted up to 28 μs time point (Fig 5C). Thus, we can infer that the pivoting motion still stands out when changing from one inactive TMD structure to another one. In addition, the pivoting point involved L768-T771, F775-W778, W778-L779, and W778-L782 pairs in common at the time of the pivoting motion.

Thus, we propose that the pivoting motion is essential for proper helix rotation toward deactivating TMD and interconverting between different inactive TMD forms. Our CG MD simulation results from the multiple trajectories indicate that the crossing angle needs to change by larger than ~45 degrees for inducing meaningful helix rotations. In addition, from the 25 trajectories, we observed that the conversion from the active to the inactive forms occurred in ~0.2 μs at the fastest and ~5 μs at the slowest. Of course, considering that a CG model tends to have less mechanical friction than the corresponding all-atom model, we should note that the actual timescale will be much slower than this simulation timescale.

From the viewpoint of microscopic reversibility, we suggest that the pivoting motion is also important in TMD activation of VEGFR-2. The fact that pivoting motion was suggested to be essential in the activation process of the TMD region of some RTKs [42] also supports our argument. Again, as observed in our inactivation process, the pivoting motion will be essential for the TMD helix to rotate by an appropriate angle around its axis for the activation. Let us particularly consider the process of changing from the inactive TMD structure corresponding to the FES region 1 in Fig 4A to the active TMD structure. During this process, the distance between the two helices and the Δ*d* value inevitably change. Subsequently, the interhelical interaction arises and a pivoting motion is favored toward overcoming any hindrance. If the pivoting motion takes place to increase the crossing angle up to ~45 degrees, the helices can relatively freely rotate such that the interface of the two helices changes by ~180 degrees. Upon close inspection, we observed that only one helix rotated when the helices interconverted their conformations between a left-handed and a right-handed ones. Namely, over the course of a handedness conversion involving a large change in the crossing angle, one helix rotated by ~180 degrees around its own helix axis. After that, TMD underwent another pivoting motion and the remaining helix rotated. In 23 out of the 25 trajectories, a free energy global minimum such as region 1 was found, and a similar inactivation mechanism was observed over which the active TMD changed to the inactive form that corresponded to this minimum region (S5 and S6 Figs in S1 File).

As we discussed earlier, the activation mechanism of VEGFR-2 TMD has not been elucidated in a detailed manner due to experimental limitations. Our simulation results revealed that a pivoting motion is important for the activation and that a significant change in the crossing angle should be accompanied. The same behavior will apply to the inactivation, and it is likely that a similar process will be observed in other RTKs bearing TMD units.

### Structural diversity of the inactive form

**Fig 7:**
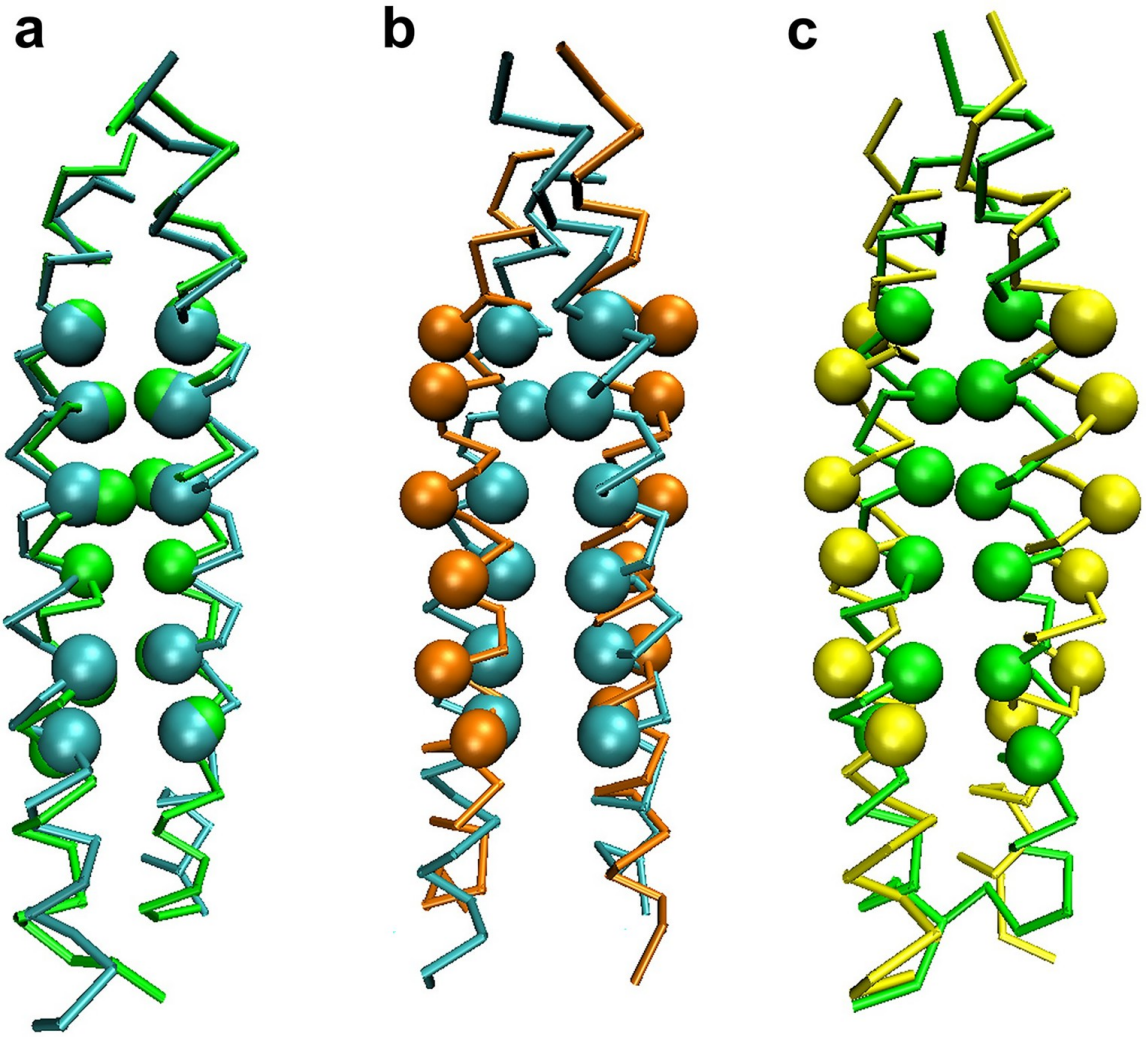
Superposition of the inactive and the active structures. (a) Superposition of the NMR (cyan) and the simulated (green) structures of inactive TMD. The traces represent the backbones and the spheres represent the residues L768, T771, A775, F778, L782, and I785, which form the interhelical contacting pairs in TMD. (b) Superposition of the NMR structures of the inactive (cyan) and the active (orange) TMD. (c) Superposition of the simulated structures of the inactive (green) and the active (yellow) TMD.

Now, let us analyze more details on the structural aspects of TMD. Toward this end, we first performed the RRCS analysis on the inactive TMD NMR structure to find interhelical residue pairs formed between helix A and helix B. The scores of 30 closely interacting pairs are listed in S1 Table in S1 File by considering residues 765–789. These residues were selected after excluding the ones that corresponded to the N-terminal and C-terminal loops of TMD. Similarly, we obtained RRCS for the TMD structures from CG MD simulations, and we detected that some structures displayed 80% or better agreement in terms of the list of highly interacting interhelical pairs. In addition, the experimental structure belonged to the region 3 of Fig 4A, and the majority of the simulated structures displaying more than 25 highly interacting pairs in common with the experimental inactive TMD structure also belonged to the same region. Although the TMD structure corresponding to region 3 is about 10% of the total, since the structural difference between region 1 and region 3, where most of the TMD structures are located, is not large, it is not unreasonable for the TMD of region 3 to represent the inactive TMD structure (Fig 4). To further confirm this, we collected TMD structures having more than 25 interhelical residue pairs in common with the experimental list. When the crossing angle, lateral helix separation, and helix rotational angles of these structures were averaged, they were quite agreeing with the values from the experimental data (Table 1). The simulated TMD structure with the highest overlap with the experimental one in terms of the residue pair list (S2 Table in S1 File) also shows quite a high level of structural similarity (Fig 7A). Also, through RRCS, we detect that I767, L768, T771, F778, W779, L781, L782, and I785 play an important role in the formation of the interhelical pairs in the inactive TMD. Experimentally, it is known that both helix A and helix B rotate by ~180 degrees along the helical axes when TMD is activated (Fig 7B) [7]. Our simulation results indeed reflect this aspect very well (Fig 7C).

## Conclusions

TMD plays an important role in the activation of RTKs including VEGFR-2. The structural change created by the binding of ligand to the ECD region activates TMD connected to ECD, and the subsequent structural change of TMD causes another change in ICD connected to TMD. This is the course of VEGFR-2 activation, and here we revealed that the nature of the activation pathway accompanying the rotations of the two TMD helices. We conducted simulations over several milliseconds using CG MD and found that the change in the crossing angle between the two helices was a key. Specifically, the two helices need to involve pivoting motion involving the crossing angle change by up to ~45 degrees, and interconversions between a left-handed structure and a right-handed one were often observed. Namely, for the two TMD helices to rotate properly to specific directions, a pivoting motion that involved a change from one handedness to the other one and then a subsequent change back to the first handedness was observed as essential. We also showed that the active TMD state without any stabilizing mutation was unstable while the inactive TMD was very stable as a somewhat diverse ensemble. Although we observed these pivoting motions by adopting inactivation trajectories, we expect that the same pivoting motion will also be essential for the activation process. The stability of inactive TMD demonstrates that spurious activation of VEGFR-2 without any binding ligand will not be likely. Ultimately, the inactivation / activation mechanism of VEGFR-2 that we have revealed will also help explain the structural changes associated with ECD and/or ICD. We also hope that our results can be useful for understanding the TMD interactions in other RTKs.

## Supporting information

S1 File Supporting information–contains all the supporting tables and figures. (ZIP)

S2 File (DOCX)
