# Supplementary material for: Elucidating activation and deactivation dynamics of VEGFR-2 transmembrane domain with coarse-grained molecular dynamics simulations
Source: PLoS One. 2023 Feb 16;18(2):e0281781. doi: 10.1371/journal.pone.0281781 (PMC9934429; doi:10.1371/journal.pone.0281781)
Supplement: S1 File — (ZIP) [file pone.0281781.s001.zip › S1_Fig.pdf]

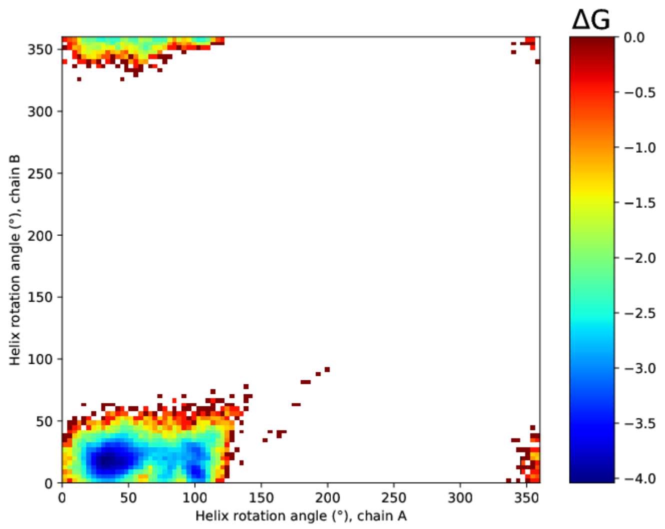

S1 Fig. Free energy profile of TMD with trajectories initiated from the inactive conformation.

Free energy landscape as a function of the TM helix dimer rotational angles when the simulation was initiated from the inactive form. The helix rotation angles of the initial structure right after the energy minimization were adopted as the zero references of the angles.
