# Supplementary material for: Elucidating activation and deactivation dynamics of VEGFR-2 transmembrane domain with coarse-grained molecular dynamics simulations
Source: PLoS One. 2023 Feb 16;18(2):e0281781. doi: 10.1371/journal.pone.0281781 (PMC9934429; doi:10.1371/journal.pone.0281781)
Supplement: S1 File — (ZIP) [file pone.0281781.s001.zip › S1_Table.pdf]

S1 Table. Interhelical residue-residue contact scores (RRCSs) of 2M59.

Helix A	Helix B	RRCS
765 ILE	764 GLU	5.26
767 ILE	768 LEU	44.59
768 LEU	764 GLU	6.17
768 LEU	767 ILE	44.60
768 LEU	768 LEU	20.96
768 LEU	771 THR	1.96
771 THR	768 LEU	1.97
771 THR	771 THR	21.66
771 THR	772 ALA	0.03
771 THR	775 ALA	0.22
772 ALA	771 THR	0.033
774 ILE	775ALA	0.49
775 ALA	771 THR	0.22
775 ALA	774 ILE	0.49
775 ALA	778 PHE	2.07
778 PHE	775 ALA	2.03
778 PHE	778 PHE	17.07
778 PHE	779 TRP	17.29
778 PHE	782 LEU	2.85
779 TRP	778 PHE	17.30
781 LEU	782 LEU	40.50
781 LEU	786 LEU	0.25
782 LEU	778 PHE	2.80
782 LEU	781 LEU	40.57
782 LEU	782 LEU	11.96
782 LEU	785 ILE	19.61
785 ILE	782 LEU	19.51
785 ILE	785 ILE	25.53
785 ILE	786 LEU	9.28
785 ILE	789 VAL	3.19
