# Supplementary material for: Elucidating activation and deactivation dynamics of VEGFR-2 transmembrane domain with coarse-grained molecular dynamics simulations
Source: PLoS One. 2023 Feb 16;18(2):e0281781. doi: 10.1371/journal.pone.0281781 (PMC9934429; doi:10.1371/journal.pone.0281781)
Supplement: S1 File — (ZIP) [file pone.0281781.s001.zip › S2_Fig.pdf]

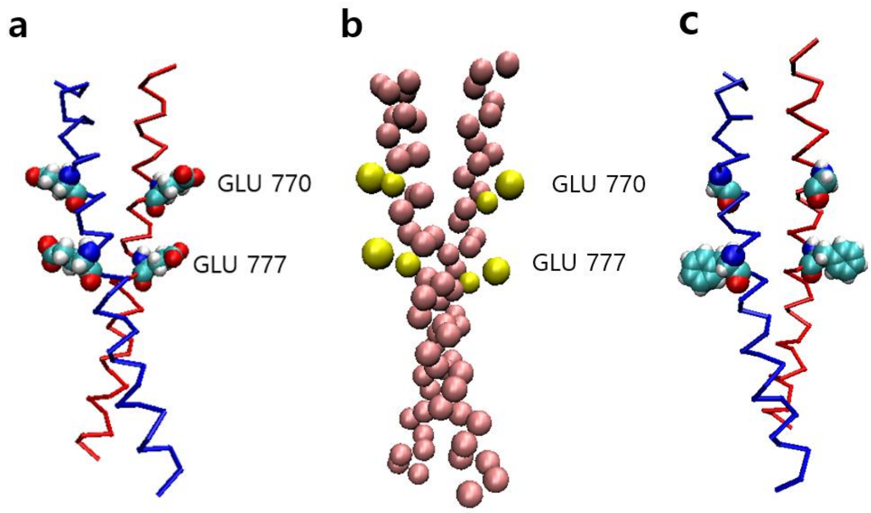

S2 Fig. Structures of mutant TMD in an inactive form versus the NMR structure of inactive TMD.

(a) Mutant TMD with the wildtype inactive TMD conformation obtained with PDB ID 2M59 and (b) its CG-represented conformation. (c) Structure of 2M59.
