# Supplementary material for: Elucidating activation and deactivation dynamics of VEGFR-2 transmembrane domain with coarse-grained molecular dynamics simulations
Source: PLoS One. 2023 Feb 16;18(2):e0281781. doi: 10.1371/journal.pone.0281781 (PMC9934429; doi:10.1371/journal.pone.0281781)
Supplement: S1 File — (ZIP) [file pone.0281781.s001.zip › S2_Table.pdf]

S2 Table. Interhelical RRCS of the inactive TMD obtained from the CG MD simulations which shows the highest concordance rate with RRCS of 2M59.

Helix A	Helix B	RRCS
765 ILE	764 GLU	0.80
767 ILE	768 LEU	43.49
768 LEU	764 GLU	5.32
768 LEU	767 ILE	44.83
768 LEU	768 LEU	28.81
768 LEU	77 THR	13.82
771 THR	768 LEU	22.63
771 THR	771 THR	47.17
771 THR	772 ALA	11.18
771 THR	775 ALA	18.51
772 ALA	771 THR	6.25
774 ILE	775 ALA	23.32
775 ALA	771 THR	0.65
775 ALA	774 ILE	40.45
775 ALA	778 PHE	14.15
778 PHE	775 ALA	15.05
778 PHE	778 PHE	33.24
778 PHE	779 TRP	14.99
778 PHE	782 LEU	22.44
779 TRP	778 PHE	39.88
781 LEU	782 LEU	2.36
782 LEU	781 LEU	3.76
782 LEU	782 LEU	61.03
782 LEU	785 ILE	35.44
785 ILE	785 ILE	0.13
785 ILE	786 LEU	56.12
785 ILE	789 VAL	2.80
