# Supplementary material for: Elucidating activation and deactivation dynamics of VEGFR-2 transmembrane domain with coarse-grained molecular dynamics simulations
Source: PLoS One. 2023 Feb 16;18(2):e0281781. doi: 10.1371/journal.pone.0281781 (PMC9934429; doi:10.1371/journal.pone.0281781)
Supplement: S1 File — (ZIP) [file pone.0281781.s001.zip › S3_Fig.pdf]

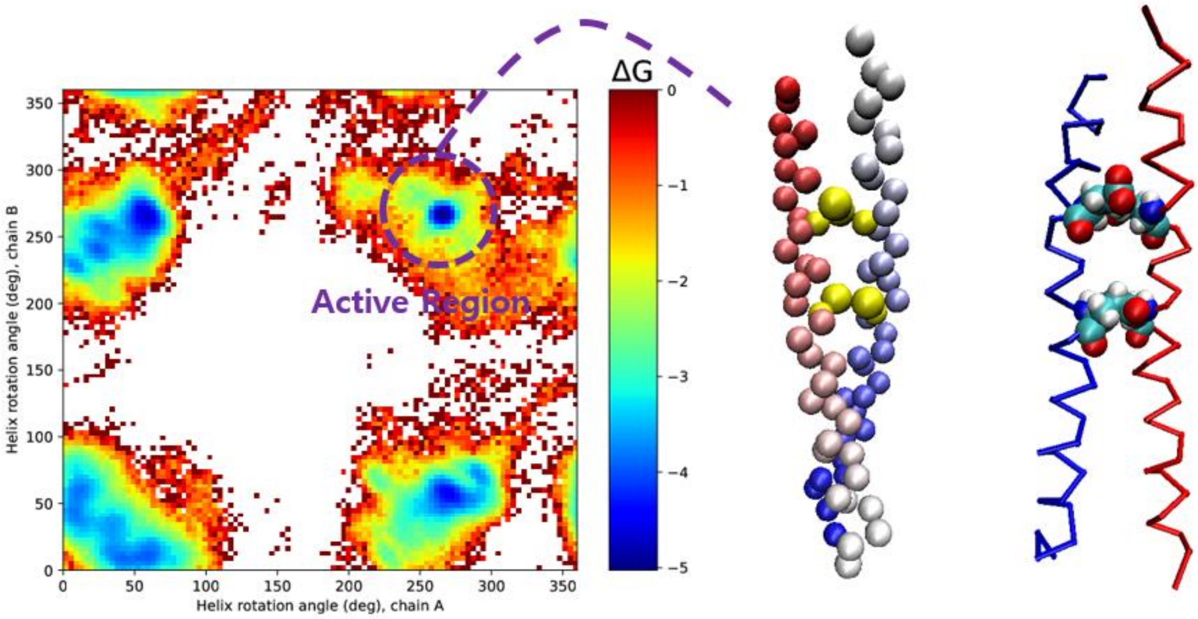

S3 Fig. Free energy landscape of the mutant TMD together with its active form structure. Free enery profile of the mutant and a representative structure of the dominant basin (left sturcture) extracted from the simulated trajectories. The active region is clearly observed in the free energy profile, which is consistent with the experimental results that showed high activity with the mutant TMD. The experimental strucutre of the mutant (PDB ID: 2MEU) is shown for a comparison (right structure). The CG MD simulation protocol was the same as the other CG MD protocols used in the main text, except that 100 μs of 10 production simulations were performed with TMD structures extracted at every 4 ns for generating the free energy surface.
