# Supplementary material for: Elucidating activation and deactivation dynamics of VEGFR-2 transmembrane domain with coarse-grained molecular dynamics simulations
Source: PLoS One. 2023 Feb 16;18(2):e0281781. doi: 10.1371/journal.pone.0281781 (PMC9934429; doi:10.1371/journal.pone.0281781)
Supplement: S1 File — (ZIP) [file pone.0281781.s001.zip › S5_Fig.pdf]

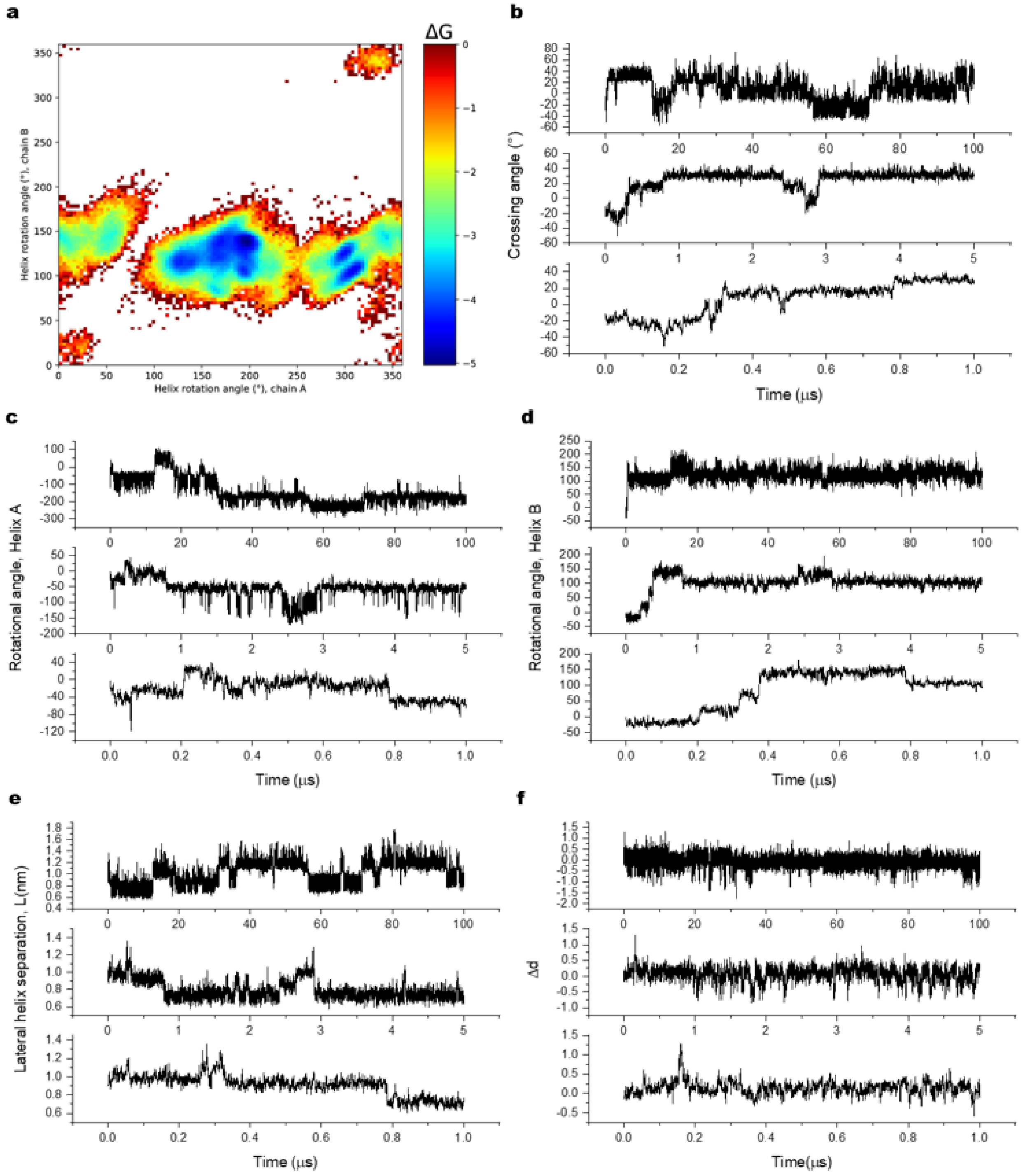

S5 Fig. FES showing the free energy minima corresponding to the inactive TMD structures and time profiles of TM helices interactions.

(a) Free energy profile, (b) time evoluation of the crossing angle, (c/d) time evoluation of the helix rotational angle of chain A / B, (e) time evolution of the lateral helix separation, and (f) the time evolution of Δ*d*, obtained from a trajectory chosen differently from the one in the main text.
