# Supplementary figures and images for: Elucidating activation and deactivation dynamics of VEGFR-2 transmembrane domain with coarse-grained molecular dynamics simulations
Source: PLoS One. 2023 Feb 16;18(2):e0281781. doi: 10.1371/journal.pone.0281781 (PMC9934429; doi:10.1371/journal.pone.0281781)

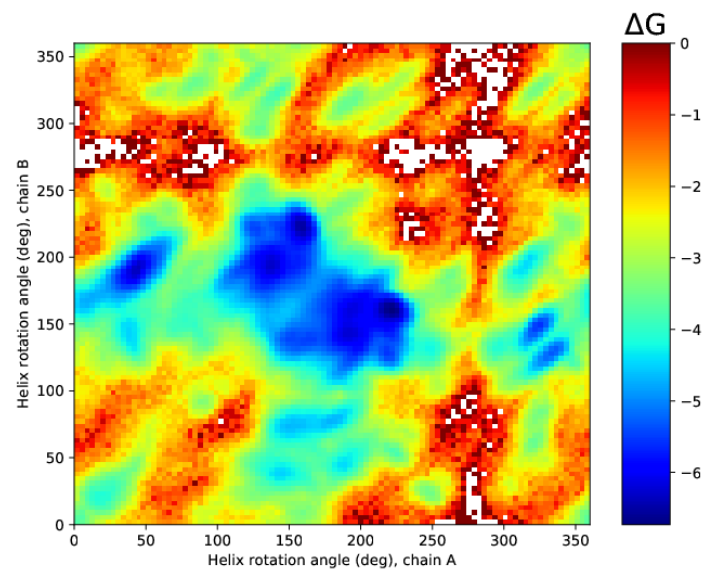

**S4 Fig. Composite FES obtained by using all 25 trajectories, started from the active TMD structure.**

Supplement: S1 File — (ZIP) [file pone.0281781.s001.zip › S4_Fig.pdf]

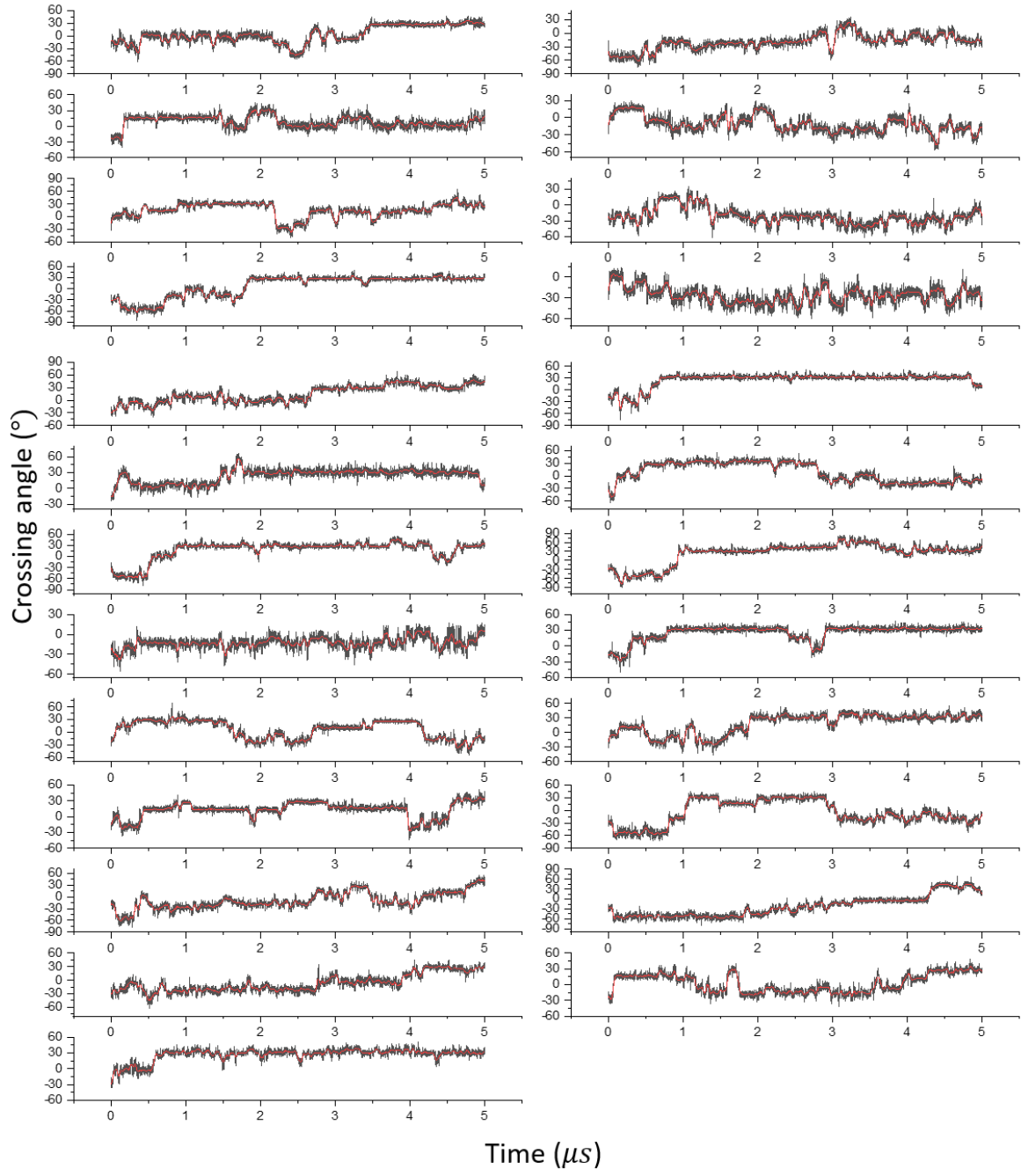

**S6 Fig. Time evolution of the crossing angle of TMD extracted from 25 trajectories.**

Supplement: S1 File — (ZIP) [file pone.0281781.s001.zip › S6_Fig.pdf]
