# Supplementary material for: Elucidating activation and deactivation dynamics of VEGFR-2 transmembrane domain with coarse-grained molecular dynamics simulations
Source: PLoS One. 2023 Feb 16;18(2):e0281781. doi: 10.1371/journal.pone.0281781 (PMC9934429; doi:10.1371/journal.pone.0281781)
Supplement: S2 File — (DOCX) [file pone.0281781.s002.docx]

**Supporting information for**

**Elucidating activation and deactivation dynamics of VEGFR-2 transmembrane domain with coarse-grained molecular dynamics simulations**

Yeonju Go^1^, Mahroof Kalathingal,^1,2^ and Young Min Rhee^1*^

^1^ Department of Chemistry, Korea Advanced Institute of Science and Technology (KAIST), Daejeon 34141, Korea

^2^ Department of Chemistry, Pohang University of Science and Technology (POSTECH), Pohang 37673, Korea


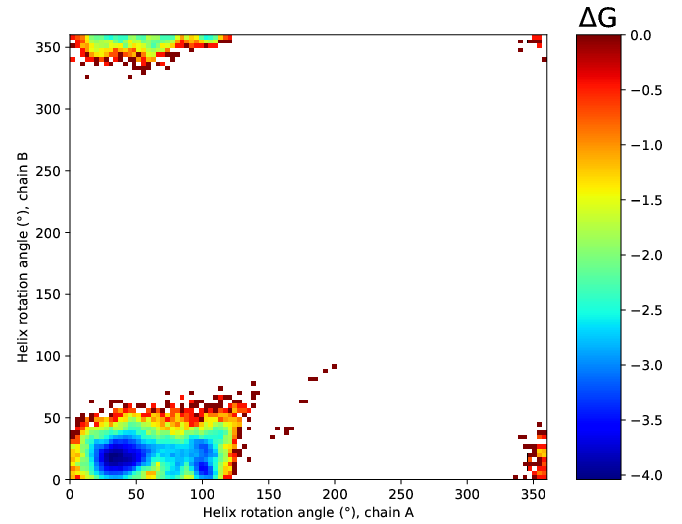


**S1 Fig. Free energy profile of TMD with trajectories initiated from the inactive conformation.**

Free energy landscape as a function of the TM helix dimer rotational angles when the simulation was initiated from the inactive form. The helix rotation angles of the initial structure right after the energy minimization were adopted as the zero references of the angles.


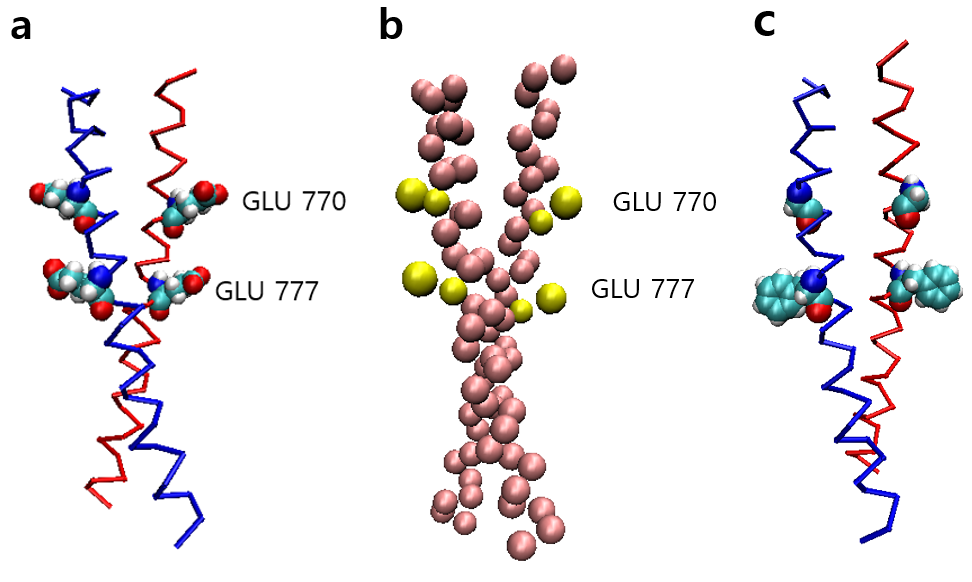


**S2 Fig. Structures of mutant TMD in an inactive form versus the NMR structure of inactive TMD.**

(a) Mutant TMD with the wildtype inactive TMD conformation obtained with PDB ID 2M59 and (b) its CG-represented conformation. (c) Structure of 2M59.


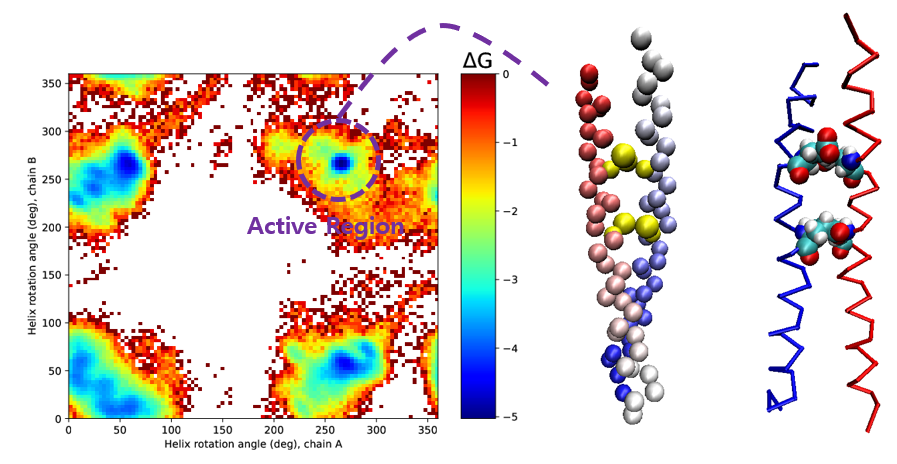


**S3 Fig. Free energy landscape of the mutant TMD together with its active form structure.**

Free enery profile of the mutant and a representative structure of the dominant basin (left sturcture) extracted from the simulated trajectories. The active region is clearly observed in the free energy profile, which is consistent with the experimental results that showed high activity with the mutant TMD. The experimental strucutre of the mutant (PDB ID: 2MEU) is shown for a comparison (right structure). The CG MD simulation protocol was the same as the other CG MD protocols used in the main text, except that 100 μs of 10 production simulations were performed with TMD structures extracted at every 4 ns for generating the free energy surface.


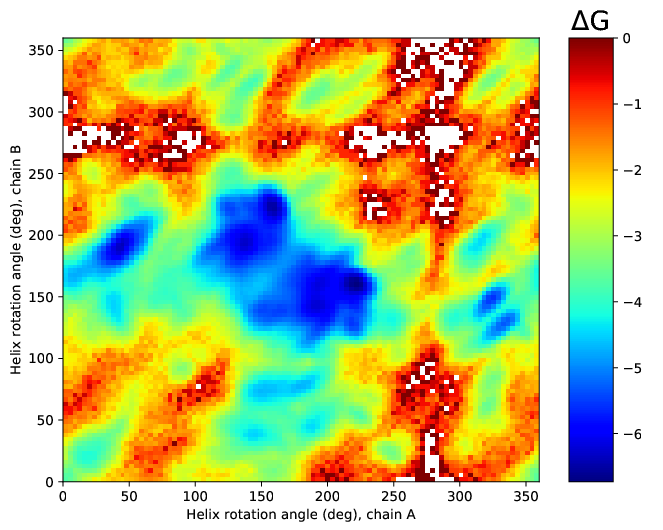


**S4 Fig. Composite FES obtained by using all 25 trajectories, started from the active TMD structure.**


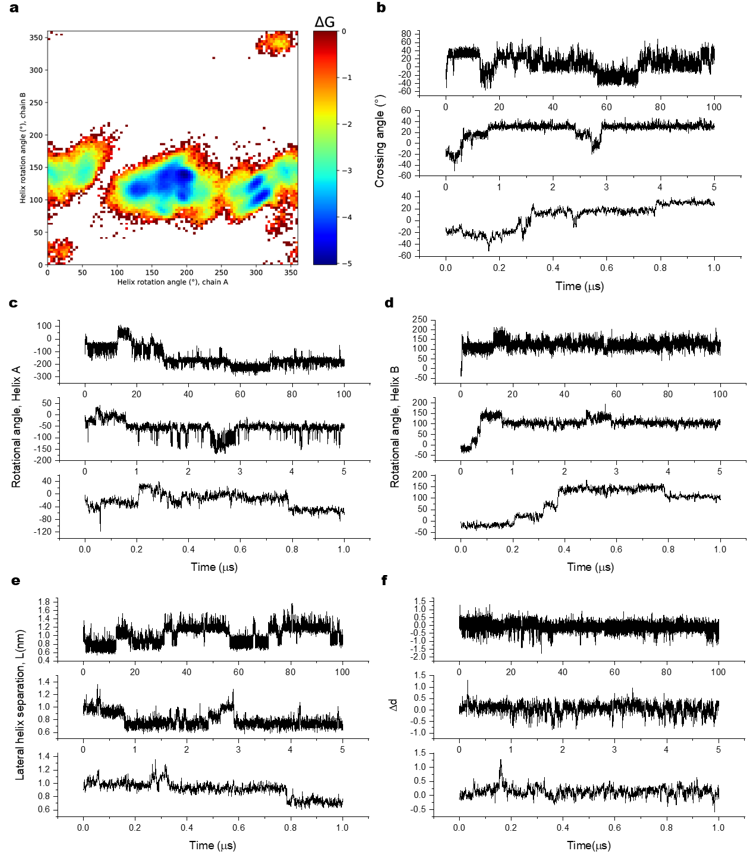


**S5 Fig. FES showing the free energy minima corresponding to the inactive TMD structures and time profiles of TM helices interactions.**

(a) Free energy profile, (b) time evoluation of the crossing angle, (c/d) time evoluation of the helix rotational angle of chain A / B, (e) time evolution of the lateral helix separation, and (f) the time evolution of Δ*d*, obtained from a trajectory chosen differently from the one in the main text.


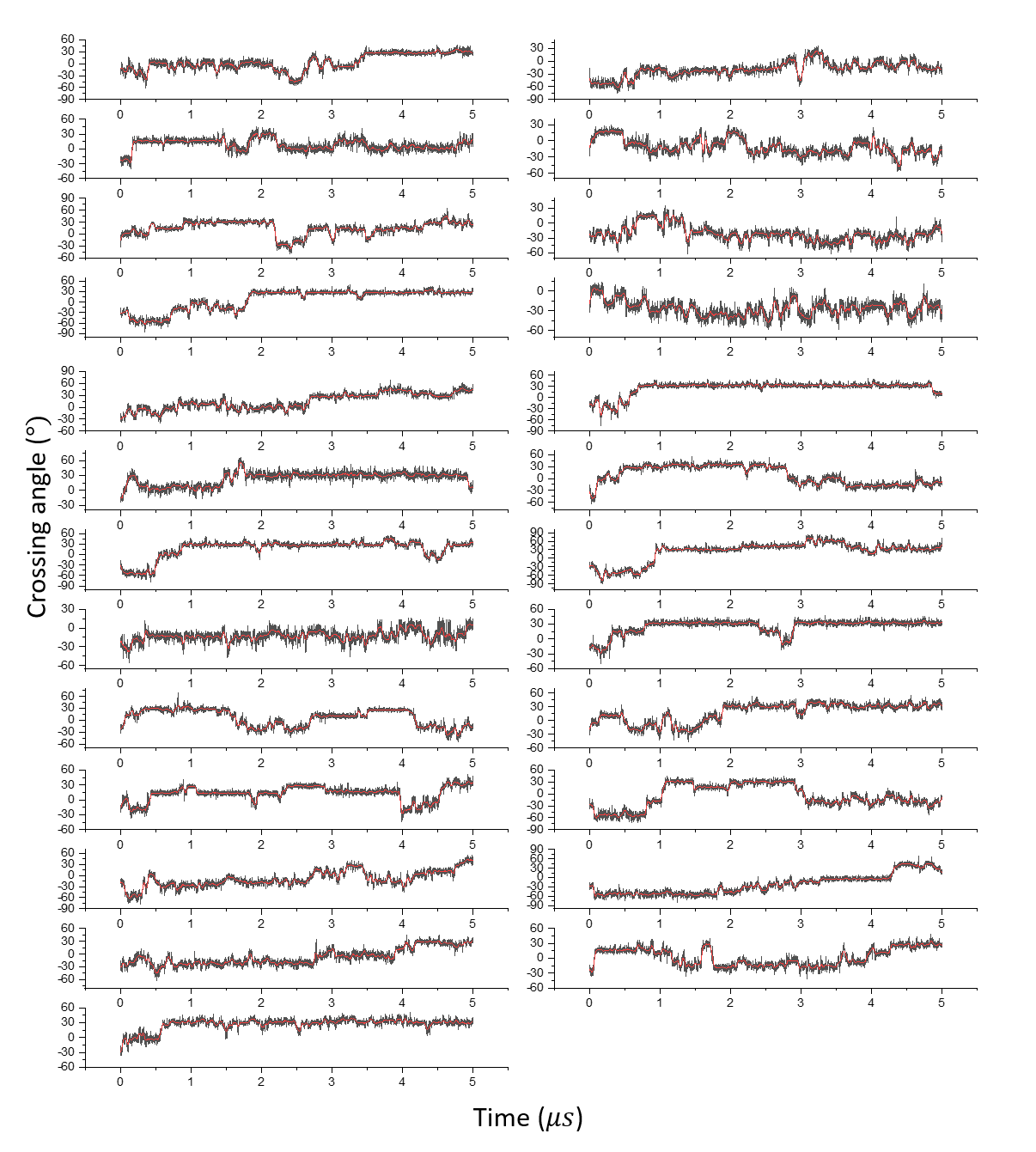


**S6 Fig. Time evoluation of the crossing angle of TMD extracted from 25 trajectories.**

**S1 Table. Interhelical residue-residue contact scores (RRCSs) of 2M59.**

| Helix A | Helix B | RRCS |
| --- | --- | --- |
| 765 ILE | 764 GLU | 5.26 |
| 767 ILE | 768 LEU | 44.59 |
| 768 LEU | 764 GLU | 6.17 |
| 768 LEU | 767 ILE | 44.60 |
| 768 LEU | 768 LEU | 20.96 |
| 768 LEU | 771 THR | 1.96 |
| 771 THR | 768 LEU | 1.97 |
| 771 THR | 771 THR | 21.66 |
| 771 THR | 772 ALA | 0.03 |
| 771 THR | 775 ALA | 0.22 |
| 772 ALA | 771 THR | 0.033 |
| 774 ILE | 775ALA | 0.49 |
| 775 ALA | 771 THR | 0.22 |
| 775 ALA | 774 ILE | 0.49 |
| 775 ALA | 778 PHE | 2.07 |
| 778 PHE | 775 ALA | 2.03 |
| 778 PHE | 778 PHE | 17.07 |
| 778 PHE | 779 TRP | 17.29 |
| 778 PHE | 782 LEU | 2.85 |
| 779 TRP | 778 PHE | 17.30 |
| 781 LEU | 782 LEU | 40.50 |
| 781 LEU | 786 LEU | 0.25 |
| 782 LEU | 778 PHE | 2.80 |
| 782 LEU | 781 LEU | 40.57 |
| 782 LEU | 782 LEU | 11.96 |
| 782 LEU | 785 ILE | 19.61 |
| 785 ILE | 782 LEU | 19.51 |
| 785 ILE | 785 ILE | 25.53 |
| 785 ILE | 786 LEU | 9.28 |
| 785 ILE | 789 VAL | 3.19 |

**S2 Table.** **Interhelical RRCS of the inactive TMD obtained from the CG MD simulations which shows the highest concordance rate with RRCS of 2M59.**

| Helix A | Helix B | RRCS |
| --- | --- | --- |
| 765 ILE | 764 GLU | 0.80 |
| 767 ILE | 768 LEU | 43.49 |
| 768 LEU | 764 GLU | 5.32 |
| 768 LEU | 767 ILE | 44.83 |
| 768 LEU | 768 LEU | 28.81 |
| 768 LEU | 77 THR | 13.82 |
| 771 THR | 768 LEU | 22.63 |
| 771 THR | 771 THR | 47.17 |
| 771 THR | 772 ALA | 11.18 |
| 771 THR | 775 ALA | 18.51 |
| 772 ALA | 771 THR | 6.25 |
| 774 ILE | 775 ALA | 23.32 |
| 775 ALA | 771 THR | 0.65 |
| 775 ALA | 774 ILE | 40.45 |
| 775 ALA | 778 PHE | 14.15 |
| 778 PHE | 775 ALA | 15.05 |
| 778 PHE | 778 PHE | 33.24 |
| 778 PHE | 779 TRP | 14.99 |
| 778 PHE | 782 LEU | 22.44 |
| 779 TRP | 778 PHE | 39.88 |
| 781 LEU | 782 LEU | 2.36 |
| 782 LEU | 781 LEU | 3.76 |
| 782 LEU | 782 LEU | 61.03 |
| 782 LEU | 785 ILE | 35.44 |
| 785 ILE | 785 ILE | 0.13 |
| 785 ILE | 786 LEU | 56.12 |
| 785 ILE | 789 VAL | 2.80 |
